# Dental Periostitis

**Published:** 1864-05

**Authors:** J. Taft


DENTAL REGISTER OF THE WEST.

VOL. XIX.] MAY, 1864. [No. 5.

### Original Essays and Communications.

### DENTAL PERIOSTITIS.

BY J. TAFT.

The dental periosteum is far more frequently the subject of disease than that of any other bones. The dentist, in the practice of his profession, continually meets with it, and that in a great variety of phases. It frequently comes with the patient, or rather leads the patient to the dentist .It sometimes follows the operations of the dentist, and baffles his best skill to counteract and remedy it.

In dentistry, far more than any other specialty of medical practice, is this affection encountered, indeed so frequent is its occurrence, and so great is its influence, that it may be regarded as one of the prominent points in dental practice; it is one, too, that has been considered very briefly, indeed, by medical writers, and scarcely at all by them, so far as it pertains to the teeth, and hence we regard it as depending almost solely upon the dental profession, for its due elaboration.

In the order of pathological conditions, as they occur to the teeth, caries stands first, exposure and disease of the pulp next, death and decomposition of that tissue third; all this may occur and the teeth still perform their functions perfectly, next irritation and inflammation of the periosteum.

This can not occur without very materially interrupting their normal functions. And following this alveolar abscess, or suppuration of the periosteum, thus effecting a detachment of the tooth from the surrounding living tissue. Doubtless, most if not all, who have for any considerable length of time been engaged in dental practice have some knowledge of periostitus and understand something of its nature, and the ordinary local causes; but the condition is so largely influenced and modified by systemic tendencies, as perhaps to escape the *full* comprehension and appreciation of every one.

There are many grades or degrees of normal vitality and life function manifested through an almost infinite variety of organizations. But beyond all these there is a great number of pathological conditions that influence this affection.

Generally inflammation of the dental periosteum does not occur till after the death of the pulp of the tooth; sometimes, however, it does occur as a reflection, or rather an extension of an inflamed condition of the pulp and lining membrane. This occurs only in cases where there is a manifest inflammatory diathesis.

Periostitis is not more liable to occur in the periosteum of of the teeth, previous to the death of its pulp than in that of other bones. But it occurs far more readily and frequently after the loss of the internal vitality; and for two or three reasons: First, because the periostium is in so close proximity to tissue, to a large extent, without vitality; second, because it is to some extent, more than normally drawn upon for vital supply. Both of these circumstances would increase its susceptibility to irritation; so that those agents that before could make no impression, are now capable of producing very marked results; such, for instance, as deposits upon the teeth, especially beneath the margins of the gums, blows or undue pressure, rude and rough operations, but more frequently than any other, or perhaps all of these, is the accumulation of fetid matter, from decaying pulp and membrane, an exciting cause of periosteal inflammation; this passes through the foramen at the point of the fang, and then operates as a very active irritant and excitant to diseased action.

This is, doubtless, by far the most frequent cause of periostitis. Usually when a pulp dies, the decomposing matter remains confined in the cavity and canals of the fangs; it is generally very acrid and irritating in its character, and is quite sufficient to produce a diseased action in the living tissue, with which it may come in contact; and especially when we consider that when such contact occurs, the absorbents are actively at work, taking up and endeavoring to remove the offensive matter. The function of the absorbents is to take up and convey away worn out, useless and offensive material. They sometimes assume to do more than they can without injury accomplish, and such is frequently the case, when they attempt to take up and convey away the remains of a dead tooth pulp; though sometimes they will accomplish it most perfectly without the surrounding tissue suffering any inconvenience; occasionally cases are found in which the pulps have lost their vitality, become decompased and the remains wholly removed, so that the pulp chamber is left a vacuum before it has any external communication.

These cases are not very frequent, however; they occur through the energy of the absorbents, and the resisting power of the surrounding tissue, together with the comparatively non-offensive character of the disintegrated tissue, for such cavities usually emit little or no offensive odor. The ability of the absorbents to accomplish this work with impunity depends upon the degree of their vitality and the systemic influence that modify their operations.

There is sometimes a near approach to disease, which, under favorable circumstances, would pass through safely, but in which even a slight systemic derangement, or ill local influence may serve to arouse active disease. Now it must be remembered that when any tissue or organ is called upon to perform more than its normal amount of work, it is far more liable to become diseased; and here not only the absorbents but the periosteum also has more than its normal amount of work to perform in the extra nourishment it has to afford to the cementum.

Again, very frequently, when a pulp dies, a small portion will be left remaining in and near the point of the fang, in a sloughing and discharging condition; now, if the fang in the canal becomes closed, either from an untimely filling, a wooden pivot, or from any other cause, that secretion is thrown back upon the living tissue, and its current checked; the absorbents are not competent to the task of its removal; and irritation, inflammation and alveolar abscess are the usual accompaniments.

The systemic conditions that modify and influence periostitis are so numerous and various in their character, that it seems almost in vain to make an attempt to refer to them within the scope of a brief article; this, however, we will leave for another time.

